# ‘They think we’re just in God’s waiting room’: A discursive study on identity aloneness in stroke survivors

**DOI:** 10.1177/13634593241279207

**Published:** 2024-09-19

**Authors:** Lewis Jefferson, Stephen Dunne

**Affiliations:** Northumbria University, UK; Northumbria University, UK

**Keywords:** ageing and lifecourse, discourse analysis, discourse and conversation analysis, health, quality of life, social inequalities in health

## Abstract

This paper examines the rhetorical strategies used by stroke survivors to attend to identity aloneness, a phenomenon in which individuals experience a sense of disconnect from others as a consequence of identity change, for which stroke is known as an antecedent. Three stroke survivors, and their spouses, were interviewed about their stroke, social support, and experiences with loneliness and identity change. The data was transcribed using a simplified version of the Jeffersonian method and analysed using a critical discursive psychological approach. This made it possible to examine the way in which the psychological business of identity aloneness was managed in participants’ talk via discursive devices such as metaphors and category entitlement, while also leaving room to consider how broader societal discourses were drawn upon. The analysis revealed two critical ways in which participants attended to the issue of identity aloneness: (1) by crafting and occupying a position of resilience; (2) by managing the impact of the post-stroke social world on their identities. These findings offer insight into how the issue of identity aloneness is made sense of by stroke survivors in the context of a discussion with an interviewer. Finally, findings informed future directions for research, including developing a comprehensive theory of identity aloneness using a grounded theory approach and developing and validating a psychometric measure of identity aloneness to be applied in a rehabilitative setting.

## Introduction

### Loneliness and stroke

Loneliness can be understood as an unpleasant experience arising from a perceived discrepancy between an individual’s desired and actual social relationships (Peplau and Perlman, 1982). While unpleasant itself, loneliness is an antecedent to further physical and mental health issues including suppressed immune-related functions (Cole et al., 2007), depression (Theeke et al., 2012), cognitive decline (Tilvis et al., 2004) and accelerated progression of Alzheimer’s disease (Wilson et al., 2007). The observation of these sequalae serves only to elevate loneliness as a target for concern. Although anyone can experience loneliness, groups with chronic disability are more susceptible (Maguire et al., 2021; Mosher et al., 2012). One such group is survivors of stroke, whose prevalence of loneliness at 30%–44% (Byrne et al., 2022) far outweighs the Office for National Statistics’ figure for the general population at 5% (ONS, 2017). According to the Stroke Association (2023), each year in the UK, 100,000 people experience a stroke. However, mortality from stroke has reduced from 66,726 in 2001 to 36,771 in 2018, contributing to the current figure of 1.3 million stroke survivors (Stroke Association, 2023). Thus, the inarguable benefit of more people surviving stroke bears with it the burden of a greater number of individuals who are statistically more likely to experience loneliness. To improve the quality of life of these individuals, it is necessary examine the aetiology of loneliness in survivors of stroke.

The cognitive and physical antecedents of loneliness are already understood in the stroke literature. For example, aphasia, which affects approximately a third of stroke survivors (CPPE, 2022), is known to be responsible for elevated levels of distress (Hilari et al., 2010) as well as diminished social contact and engagement in social activities compared to non-aphasics (Cruice et al., 2006; Davidson et al., 2003). Mobility also impacts survivors’ ability to socialise, as mediated by fewer opportunities to travel and a shorter list of suitable activities through which to engage with others (Mukherjee et al., 2006). Not all sequelae of stroke are this well-documented in terms of their relationship with loneliness, however. Identity change is also acknowledged as a problematic element of the stroke experience (Anderson and Keating, 2016), but is one which tends to be examined in isolation to loneliness in the stroke population. More recently, a study exploring the experiences of loneliness in stroke survivors revealed that ‘identity aloneness’ was a recurring feature of survivors’ talk on the matter of loneliness (Yang et al., 2022). Identity aloneness manifested as lamenting the transition from an independent pre-stroke self to a post-stroke self dependent on the care of others and difficulties negotiating these conflicting identities. This nascent concept could provide a basis from which to understand the way in which identity may influence and shape survivors’ experiences with aloneness, and by extension the way in which they experience loneliness. Therefore, the present study aims to address the question ‘how is identity aloneness attended to by stroke survivors?’.

### Identity and aloneness

The aloneness component of identity aloneness can be understood in the context of Diekema’s (1992) framework. Unlike loneliness, which Diekema calls the ‘phenomenological experience of aloneness’, aloneness is an objectively measurable construct. It manifests temporally as interactional, involving a lack of everyday, transient interaction and relational, involving a lack of meaningful enduring connections. Socially, aloneness can be other-imposed, such as through shunning, mutually constructed, such as with privacy, or self-imposed, through escapism in substance abuse, for example. While this conceptualisation of aloneness elucidates the multiplicity of pathways to loneliness, the matter is further complicated by identity change.

Identity has been subject to various theoretical shifts over time. A prominent early view that attends to the social significance of identity has been social identity (and the related self-categorisation) theory (Tajfel and Turner, 1986; Turner et al., 1987). The fundamental axiom of social identity theory is that in contexts where group membership is salient, individuals undergo ‘depersonalisation’, identifying less as an individual and more as a member of the salient ingroup. When individuals ‘self-categorise’ as a member of the ingroup, they begin to incorporate the meanings associated with that ingroup into their identity (Turner et al., 1987). Haslam et al. (2008) found that stroke survivors who had more pre-stroke group memberships with which they were strongly affiliated had greater life satisfaction than those with fewer. This was mediated by the maintenance of these memberships through and after stroke. It is clear from this research that maintenance of group memberships constitutes a more robust social identity, which (in theory) would lead to reduced aloneness, and so it would not be unreasonable to suggest that the reported increase in the composite variable of life satisfaction would also correspond to the diminishment of loneliness.

Recently, perspectives have focussed on narrative. In particular, the life thread model (Ellis-Hill et al., 2008) conceptualises identity as a ‘life story’ constituted by a multiplicity of life threads (or stories) that exist relative to each other and extend across time. This narrative structure functions as a sense-making tool in which events and actions are structured in a symbolic way, such that the morality and motivations of actors can be understood (Ellis-Hill et al., 2000). Each life thread represents a story that individuals can tell about themselves, and these together constitute a life story/identity. As well as there being multiple life threads, their temporal dimension, which forms a trajectory extending into the future, gives individuals’ sense of self stability and coherence. The onset of stroke, then, and chronic disability more broadly, constitutes a biographical disruption (Bury, 1982), in which the predictable course of adult life, and thus identity continuity, is disrupted. At least two examples draw on this theory. First, the loss of physical function that accompanies stroke means that the ‘story’ of their previously held symbiotic relationship with their able body is cut short (Ellis-Hill et al., 2000). In turn this severed life thread, having previously supported, destabilises the survivor’s life story, forcing them to renegotiate their identity to maintain continuity. Nochi (1998) also explains how the self-narratives of traumatic brain injury survivors are negatively impacted and elucidates how the survivors are able to reframe and reconstruct them in a positive or neutral manner (Nochi, 2000).

### The present study: issues with epistemology and lens

Issues with these approaches exist in their utility for explaining identity aloneness. Social identity theory treats identity as a cognitive, pre-discursive phenomenon that lies dormant, ready to be activated (Benwell and Stokoe, 2006). It is applied within an essentialist, cognitivist paradigm, the dominant view in psychology in which latent mental processes are the object of study. Alternatively, social constructionist paradigms, such as discursive psychology, have allowed for the study of language as constitutive, rather than representative, of psychological issues. Potter (1996: 160) explains that this view of language is preferable to the problematic activity of trying to parse out language and mental representations when in reality they tend to be conceptually reliant on one other when communicated, with ideas of what mental representations are derived from what people say and vice versa. Psychological business carried out in everyday talk is thus an opportunity to study psychological matters without making ontological claims about invisible cognitive structures.

Another matter is analytical lens. Within their paper, Ellis-Hill et al. (2008) claim the life thread model is compatible with a discursive approach. However, within subsequent work citing the model (Whiffin et al., 2019; Whiffin and Ellis-Hill, 2022), the only discursive matters attended to are those of broad contextual significance. For example, passing reference is made to ‘master narratives’, such as the medical model and disability norms, which apparently govern participants’ understandings of illness are made without any kind of fine-grain analysis of participants’ discursive practices. This distinction is what Wiggins (2017) refers to as analytical or contextual lens; the degree to which attention is paid to narrower versus broader contextual factors, and the degree to which participants are seen as agentic in their talk versus bound by broader interpretative resources. Critiques are offered for both extremes; analyses relying on hypothetical discourses obscure what is really going on in the interactions by imposing the theoretical and political agendas of the analyst onto the analysis (Schegloff, 1991, as cited in Benwell and Stokoe, 2006), whereas adopting too narrow an approach is seen as producing an ‘impoverished account’ of participants’ social practices (Fairclough, 1992, as cited in Benwell and Stokoe, 2006). There is thus a demand in the stroke literature for a methodology which, while still appreciating the possibility of influence from broader discourses, takes a more narrow and detailed approach to analysis which attends mainly to how participants orient themselves in situ and construct their own social realities. The approach used here will therefore be critical discursive psychology (CDP). The present study’s aim is to use this approach to provide an answer to the question: ‘how is identity aloneness attended to by stroke survivors?’

## Methods

This project was preregistered on the Open Science Framework (OSF): https://osf.io/fwhkc. All data and materials associated with the project can be found on the OSF: https://tinyurl.com/identityaloneness.

### Approach

The present study employed the qualitative methodology CDP (Wiggins, 2017: 70–76). CDP is an epistemologically social constructionist approach capable of examining the psychological business carried out in talk and text within the context of both turns taken between speakers and the broader sociocultural backdrop. The present implementation of CDP is weighted towards talk and text analysis but considers sociocultural contexts where appropriate and justified by participants’ own orientations. A set of analytical tools which permitted an inductive analysis of the kinds of social actions carried out by speakers were utilised; the discursive devices (Wiggins, 2017: 170). Discursive devices, such as metaphors and category entitlement, are strategies used by individuals to complete social actions such as constructing accounts, managing accountability and stake and negotiating psychological issues like identity. Through examining talk in this way, it was possible to empirically demonstrate how participants attended to identity aloneness.

### Participants

Recruitment information was circulated through third-sector support organisations, stroke support groups and the researchers’ social media accounts. In total three primary participants (PPs: three males; 63–72 years old; *M_age_* = 66.67, SD_age_ = 3.86) were recruited for the project. Three secondary participants (SPs), all of whom were female and were the wives (and in two cases, primary carers) of the three PPs, provided support for the PPs as well as providing a platform for discursive nuance in the interviews. Additional data were not collected for the SPs. All participants lived in the North East of England at the time of the study. Pseudonyms were generated for PPs and SPs. Detailed participant characteristics can be found on the OSF.

Simon was a 65-year-old man who lived at home with his second wife, Karen. He had been medically retired since his stroke in 2010, which he described as a cerebral haemorrhage. Simon’s first wife cared for him around the time of his stroke, but sadly passed away afterwards. Simon’s stroke impaired his vision, memory and cognition. Simon worked as a web designer and continues to do so on a voluntary basis.

Peter was a 72-year-old man who lived at home with his wife and primary carer, Joan. He was retired, but not explicitly in a medical capacity. His first stroke occurred in 2000, and the second in 2005, which he described as a blockage. Collectively, the sequelae of these strokes amounted to impairments in vision, speech, motor control, memory and emotional stability. Peter originally trained as an engineer but retrained as a lecturer shortly before his first stroke. He does a wide range of voluntary work concerning the stroke community, including public speaking.

Terry was a 63-year-old man who lived at home with his wife and primary carer, Sarah. He had been medically retired since his strokes in 2007. These consisted of a transient ischaemic attack, followed 1 week later by a full stroke. As a result of his strokes, Terry reported having impairments in vision, motor control, attention and memory. Terry worked as a heavy goods vehicle driver before his stroke, and now enjoys spending time with his family, going on holiday and tending to his greenhouse.

### Materials

An interview schedule adapted from Yang et al. (2022) which had already investigated loneliness in stroke, guided the topics discussed in the interviews. A section on identity change was added to generate discussion concerning PP’s sense of personhood. This section was grounded in the existing literature, with particular reference to narrative theory (Ellis-Hill et al., 2008), identity continuity (Becker, 1993; Wolfenden and Grace, 2012) and biographical disruption (Bury, 1982). It was hoped that the schedule would bring together the concepts of identity change and aloneness.

The UCLA short form developed by Hughes et al. (2004) was used during the demographic data collection phase of recruitment to gain an initial understanding of participants’ loneliness. This three-item measure (α = 0.72) asked participants to rate how often they feel they ‘lack companionship’, ‘feel left out’ and ‘feel isolated’. This was chosen to gauge experiences of loneliness whilst minimising participant burden. This was especially important in stroke survivors who are prone to experiencing fatigue.

### Procedure

The project was conducted in accordance with the Declaration of Helsinki and received ethical approval from Northumbria University Ethics Committee (Ref: 3732). Online semi-structured interviews took place on Zoom and Microsoft Teams (depending on participant preference). Online interviews were chosen as part of the initial drive for nationwide recruitment and post-recruitment were still considered sufficient to address the needs to the study. Written consent was obtained prior to interview and reobtained verbally before the interview commenced. The topics on the interview schedule were cycled through in order, while also giving room for participants to extend the remit of the topics raised as they saw fit. Breaks were offered around the mid-point of the interview, and interviews were concluded when all topics on the schedule were covered and when participants had verbally confirmed that they had also been able to cover what they wanted. Interviews lasted between 40 and 50 minutes (*M* = 46.09, SD = 4.61), and were recorded for transcription.

### Procedure for analysis

The analysis followed a CDP approach (Wiggins, 2017: 70–76). First, familiarisation of the dataset occurred through transcription itself and then repeated readings of the transcripts. Transcription followed a partial version of Jefferson’s (2004) method, wherein a single-spaced font is used alongside specific notations for features of talk, such as overlapping talk, speed of talk, loudness and laughter. Second, notes were made on the transcripts which detailed the discursive devices (such as category entitlement and extreme case formulations) used by participants to achieve social actions (e.g. attending to issues of identity). These were the analytical tools used to justify and support the identified presence of identity work and aloneness construction, the social actions of interest. Finally, instances of these actions were extracted and collated under semantic level headings which formed the answers to the research question.

## Analysis & discussion

The aim of the present study was to provide an answer to the question ‘how is identity aloneness attended to by stroke survivors?’. In general, participants’ scores on the UCLA short form, as well as what was said in the interviews, suggested low loneliness levels, unlike those in the Yang et al. (2022) and Dunne et al. (2023) studies. This meant that the focus of the analysis shifted towards analysing the rhetorical strategies participants used to attend to identity aloneness, as opposed to the original analytical interest of the construction of identity aloneness. There were two key rhetorical strategies identified in the transcripts through which participants attended to identity aloneness. The first was by crafting and occupying a position of positive resilience, constructing life after stroke as something one had to ‘get on with’. Second, they did so by managing the way in which the post-stroke social arena impacted their identities, particularly the rekindling of old friendships and the lack of understanding of non-survivors. These rhetorical strategies are evidenced below.

### Participants attended to identity aloneness by crafting and occupying a position of resilience

Throughout, participants built up a version of reality in which resilience and positivity protected against experiencing identity aloneness. ‘Get on with it’ was a recurring motif across all participants. This is implicit in extract 1, wherein Peter constructs his identity change using a contrasting narrative structure.

Extract 1:


1

IV:

changed the way you experience loneliness you know on on the rare

2

occasion that you might now (.) err

3

PP:

(unclear) put into words it that (.) it’s like bereavement

4

IV:

mhm

5

PP:

my whole life has gone (.) I mourn that sometimes (.) but now

6

I’ve got a new life (1.0) a new start (.) new way of having to do

7

things (.) so I treat it that way (.) my old life was (.) what I

8

could do I don’t think about I (.) I can’t do that anymore

9

IV:

mhm

10

PP:

I just I put that to one side that’s (.) my old life (1.0) yes I

11

get sad (as you say) I do get sad sometimes (.) about it (.) but

12

now I concen- concentrate on the new life (.) what I’m doing now


The extract begins with the interviewer concluding a question about the impact of Peter’s identity change on his experience of loneliness after his stroke (ll. 1–2). In lines 3–6, Peter constructs the loss of his pre-stroke self metaphorically as a ‘bereavement’ in which his ‘whole life’ has gone and been replaced with a ‘new life’. Multiple discursive devices work together here to support this identity work. First, the use of the bereavement metaphor works to construct the category of identity loss in terms of the characteristics of mortality. According to Wiggins (2017), metaphors are non-literal, easy-to-undermine statements which function rhetorically to emphasise some features of categories (i.e. death and identity loss) and conceal others. Here, the features of loss and irretrievability associated with death are exemplified, whereas the literal distinctions between categories are glossed over. This is further strengthened with Peter’s use of the verb ‘mourn’ in line 5, as opposed to a more semantically neutral choice, such as ‘miss’.

Metaphors are often found alongside other devices, such as extreme case formulation (ECF) and category entitlement (Wiggins, 2017). ECFs are semantically extreme phrases designed to be oriented to as non-literal. This is seen in Peter’s construction of his ‘whole life’ as having been gone (l. 6). Similarly, one participant in Kirkwood et al. (2013) recounted how they went from having ‘all’ their teeth, to them being ‘fully gone’ after an attack in Glasgow. In this case the ECF works to furnish the claim of having been attacked with legitimacy (Pomerantz, 1986), supported with the use of temporally contrastive categories of having and not having teeth. In the present case, the ECF ‘whole life’ is positioned against the contrastive category ‘new life’ in line 7. This contrast is also embedded in a narrative structure in which a clear sequential order is established from old to new, thus reinforcing the credibility of Peter’s claim.

Further credibility is added to Peter’s account of identity transition by drawing on the categories of ‘what [he] could do’ in his ‘old life’ and what ‘[he] can’t do’ in his ‘new life’ (ll. 6–8). These categories bear resemblance to the contrastive cultural and medical master narratives of ‘normal’ and ‘disabled’ observed by Glintborg (2015). These discourses were argued by Glintborg to have opened up subject positions (Davies and Harré, 1990) for their participant Eric, which he resisted and instead constructed his own identity grounded in his volunteer work. In a similar vein, Peter orients to the choice between getting ‘sad sometimes’ (l. 11) and concentrating ‘on the new life’ (l. 12). He thus resists a subject position defined by what he can’t do anymore by ‘putting that to one side’ (l. 10) and constructing a version of reality in which the sequelae of stroke do not limit his available positions. This draws on the ‘agent-subject distinction’ device, which speakers tend to use to manage their accountability (Wiggins, 2017). Typically, this device is used to play down agency, such as in the case of suspect-police interrogations (Stokoe and Edwards, 2008), but here Peter uses it to upgrade his agency over his identity, adopting a position of resilience.

In summary, Extract 1 works to show how Peter positioned himself as being an active agent in constructing a positive identity who does not dwell on the past and practices acceptance of the present. This is despite the vividly negative account of the bereavement of his pre-stroke identity, which provides the necessary backdrop to situate his position of resilience. Implicit in Peter’s identity work is the ‘get on with it’ motif, which constructs progression as a necessary element of identity development. This is more explicit in Extract 2, where Terry produces an account of the events shortly after his stroke.

Extract 2:


1

PP:

aye .hh saved my life (.) s:::o=

2

SP:

=they learned you things

3

PP:

they learned us to walk again cause (.) I couldn’t walk (.) I

4

couldn’t shave (.) and I I’m partially sighted now I lost (.)

5

s some of the peripheral vision on my eyes (1.0) [sooo

6

IV:

    mhm]

7

PP:

they took my driving license off us (.) which I didn’t like

8

(.) but I cant drive anymore s::o (.) but you’ve just got to

9

crack on

10

IV:

aye just doing the best you can

11

PP:

aye

12

IV:

mhm

13

PP:

I’m like that Lewis (.) I don’t like to be beaten

14

IV:

[no

15

PP:

nah ] I couldn’t sit and cogitate s::o (.) I just crack on get

16

on with it


Extract 2 begins with Terry’s appraisal of the National Health Service’s (NHS) role in saving his life (l. 1). In line 2, Sarah initiates an account that Terry was not only saved by the NHS but was also taught things. Terry’s following turn lists walking (l. 3), shaving (l. 4), but also the loss of his driving licence (l. 7). Three-part lists are a common feature of talk which work towards making statements seem more factual (Wiggins, 2017), and are often finished with generalised list completers when a third specific subject is not available (Jefferson, 1990). However, the list that Terry produces, which functions rhetorically to build credibility and ‘factualise’ Sarah’s initial utterance, does not rely on a generalised list completer. Instead, it is used to shift subject and emotion category from gratitude for the NHS to dissatisfaction with ‘them’, who ‘took [his] driving licence off [him]’, which he ‘didn’t like’ (l. 7). This shift is possible because the subject constructed is of nebulous quality; it morphs from the NHS into a seemingly homogenous ‘they’, which is then deployed as the category responsible for the loss of Terry’s driving licence. Similar rhetoric has been observed in the discourse of those speaking out against refugees (Condor et al., 2006; Hanson-Easey et al., 2014), where the ‘us versus them’ trope functions to diminish the complexity of social categories so that certain claims can be made. Psychological essentialism theory (Rothbart and Taylor, 1992) refers to this as entitativity; the belief that some groups of social categories share a ‘unitary, homogenous character’. In the present case, Terry’s category work constructs both the NHS and (presumably) the Driver and Vehicle Licencing Agency (DVLA) as a singular, homogenous category which both taught him things but took something from him.

The assessment/second assessment device is also used here. Assessments make a judgement or appraisal about something and can be objective or subjective (Wiggins and Potter, 2003). Second assessments in turn follow a preference structure (Pomerantz, 1984), meaning they match the initial assessment’s evaluative direction. Thus, second assessments which disagree with initial assessments must be managed carefully (e.g. with disclaimers) so as to not break the preference structure. Terry achieves this by shifting the definitions of the category of focus from the NHS to ‘they’, reframing the discussion away from his assistance from the state towards the way in which it has hindered him. This also constitutes a novel way of maintaining the preference for agreement (Wiggins, 2017).

It is well established that removal of an individual’s licence to drive on medical grounds is a major cause of independence loss and thus identity disruption (Sanford et al., 2019), as well as diminished social engagement and social circle size (Chihuri et al., 2016). Additionally, Yang et al. (2022) revealed that diminished independence was a contributor of identity aloneness and thus loneliness. Although not explicit in Extract 2, a pertinent interpretation would root Terry’s dislike of ‘their’ actions (l. 7) in its impact on his independence, and thus sense of self and state of aloneness. Terry then goes on to restate that he ‘can’t drive anymore s::o (.) but you’ve just got to crack on’ (ll. 8–9). Note that ‘crack on’ is a colloquialism for ‘get on with it’ in England’s North East dialect. Similar to Peter’s positioning work in Extract 1, two positions are offered up in the course of Terry’s category work that reflect an agent-subject distinction. First, the position offered to Terry by ‘them’ in which his identity is controlled to some degree by what ‘they’ can take away from him, but also an agentic position in which he can decide to ‘crack on’ and continue to develop a positively oriented identity despite the adversity of losing his driving licence. He chooses to adopt this position, as evidenced by line 10, in which he explicitly declares ‘I’m not like that Lewis (.) I don’t like to be beaten’ and on lines 12–13, where he claims that he ‘couldn’t just sit and cogitate’. These utterances are rhetorically strengthened with the use of the subjective assessment (‘I don’t like’), through which Terry orients to this preference as his own. This avoids accusations that he is making any morally loaded claim about what others should do (subjective vs category assessments; Wiggins and Potter, 2003). Also, in deploying the modal verb ‘couldn’t’, Terry constructs the passive subject position as something that is not, and could never be, an option for him.

Overall, these two extracts demonstrate some of the ways in which participants attended to identity aloneness by crafting and then occupying a position of resilience. This was assisted implicitly in Extract 1, and explicitly in Extract 2 by the ‘get on with it’ motif which was observed throughout the dataset.

### Participants attended to identity aloneness by managing the impact of the social world on their identity

Participants also made relevant the way in which the changes in their social reality after stroke had positive or problematic effects on their identity, and thus experience of identity aloneness. In Extract 3, Simon orients to the rekindling of friendships which he attributes as a positive consequence of his stroke.

Extract 3:


1

PP:

erm (.) I dunno there’s a there’s a thing (1.0) a positive s- well

2

(.) err I mean apart from retiring early on a full £pension£ heh

3

.hh erm (.) that’s that’s just obviously from a financial point of

4

view (.) erm I’ve never thought about positives before .hh I think

5

I was thinking about things that I cant do any more

6

IV:

yeah

7

PP:

erm (.) I mean do you find people giving you yes there’s positives

8

out of it (.) I mean suppose one one thing I suppose (one ways) is

9

the rekindling of friendships (.) I had (.) I m- mean the golf

10

buddies I had

11

(.) I have now erm I knew erm the guy I told you he’s selling his

12

business (.) erm (.) he’s I knew him at college (.) so I was at

13

college with him (.) erm different courses obviously (.) err bud I

14

played badminton with him and well you know we used to be in the

15

same social group there (.) and then we- I got married and he (.)

16

we went into our bus- our careers and things and lost touch with

17

him (.) erm till quite recently erm when he’s actually actually

18

it’s a    funeral heh .hh erm where (.) erm in fact it might

19

have been (name)’s funeral my wife’s funeral (.) erm I met him

20

then again decided to you know to carry on (sort of things) so (.)

21

things you know kind of friendships kind of getting re- remade I

22

suppose .hh erm

23

((6 lines omitted))

24

PP:

and years .hh so yeah its erm (.) that’s a positive that’s come

25

out of- whether that’s come out of the stroke or whether that’s

26

just come out of (.) I suppose in a way it has because it’s a it’s

27

the kind of the social kind of (.) arena is different now because

28

I’m=

29

IV:

=mhm=

30

PP:

=I wouldn’t have I wouldn’t have done things that I .hh you know I

31

   better get this right I would have would have done things

32

differently had I not been disabled heh (you know)


Extract 3 begins after the interviewer asked whether there were any positives that came out of Simon’s stroke. In line 1 Simon introduces uncertainty with ‘erm (.) I dunno there’s a there’s a thing’, followed by a pause (‘1.0’). In doing so, he initially treats the issue of positivity as somewhat incompatible with stroke. The hesitative ‘erm’ preceding the main utterance as well as the following one-second pause draws on what Wiggins (2017) refers to collectively as ‘silences, pauses and hesitations’. Jefferson (1989) suggested that when silence appears it indicates trouble in the interaction. This is resolved with the utterance ‘err I mean apart from retiring early on a full £pension£ heh’ (l. 2), which is treated as a joke and a non-genuine answer by Simon himself. This is achieved by using the phrase ‘apart from’, as well as the use of ‘smiley voice’ (Jefferson, 2004) combined with an affect display of laughter. Affect displays are a device used to manage psychological business, such as the use of ‘mmm’ to make relevant gustatory pleasure at mealtimes (Wiggins, 2002); here it allows his pension remark to be treated as a tongue-in-cheek response. Indeed, Simon declares his pension remark as ‘obviously’ just one component of his post-stroke life: ‘a financial point of view’ (l. 3). By doing this, he also makes relevant the existence of other perspectives through which positivity can be understood in relation to stroke. However, Simon does not then go on to list these. Instead, he declares that he has ‘never thought about positives before’, and that he was thinking about ‘things [he] can’t do’ (ll. 3–5). The introduction of narrative works to account for being unable to answer the interviewer’s question. A wider analytical lens might interpret this as drawing on a broader ‘materialist’ interpretative repertoire, in which it is ‘common sense’ (Billig, 1991) to attend to issues concerning finance but not, say, issues of social, psychological or spiritual significance. Similarly, a medical-model repertoire may be constraining Simon’s discourse to the pathologisation of stroke. This parallels findings in Glintborg (2015), in which it was demonstrated that the participant’s identity was co-constructed as disabled by his physician. Glintborg argued that this kind of talk constrained the participant’s available discursive resources to what his acquired brain injury took away from him, denying him the agency to construct his own identity. This is apparent in Simon’s account of never having thought of positives (l. 4). Finally, Simon initiates an account in which ‘there’s positives out of it’ (ll. 7–8).

The next section of the extract is embedded in a narrative account of how Simon’s friendship was rekindled, some of which is not directly relevant to the present analysis, hence the omission. The account is initially prefaced in line 8 with hedging (‘I suppose’), which functions to make tentative the claim that ‘the rekindling of friendships’ (l. 9) is indeed a positive outcome of stroke. Hedging is often used in this manner, and helps to manage a speaker’s accountability by avoiding making a specific claim about something and provides the option to retract under contestation (Wiggins, 2017). In lines 9–15 Simon recounts the timeline of his friendship with his ‘golf buddies’ (l. 9). This narrative structure elevates the account’s credibility (Potter, 1996) as the events therein are both anchored in sequence and implicitly to each other. Notably, Simon treats the implicit nature of these events’ relatedness as common sense. For example, he accounts for knowing his friend by invoking their shared social category memberships (having went to college and having played badminton together; ll. 11–14), as well as accounting for drifting apart by invoking natural life events that ‘happened’ (his marriage and going into his ‘career and things’; ll. 14–15). Thus, instead of having to manage these events in terms of the agency of the people involved, the narrative serves to embed these events in a sequence through which the people involved are passive recipients to the unfolding of time. The narrative is ended in lines 15–20 as he accounts for the decision ‘to you know to carry on (sort of things)’ as being a result of meeting again at his first wife’s funeral. Thus, a contrast is formulated in which the undoubtedly sad occasion of his wife’s funeral is juxtaposed with a positive remaking of a friendship, the significance of which is oriented to by Simon line 16; ‘actually actually it’s a funeral heh’.

In the final section of the extract, Simon produces a reflective account concerned with the degree to which his stroke is accountable for the rekindling of his friendship. His deliberation (‘whether that’s come out of the stroke or whether that’s just come out of (.)’; ll. 22–23) is resolved with the claim that ‘the kind of the social . . . arena is different now’ (ll. 23–24). That the social arena has changed is explicitly positioned as a result of the stroke (‘I suppose in a way it has’; l. 23) and contrasted against an alternative timeline; ‘I . . . would have done things differently had I not been disabled heh’ (ll. 27–28). This latter contrastive claim is managed carefully with the use of vagueness (as opposed to detailing alternative actions) as well as the use of a modal verb in the preceding talk ‘I better get this right’ (ll. 26–27). Vagueness is often used to manage stake in a particular claim (Potter, 1996), and modal verbs are used to make obligations relevant in an interaction (Wiggins, 2017). Since Simon has already attended to his own category membership (disabled) in making the claim about the differentness of his social arena, using knowledge drawn from a category to which he does not belong (not disabled) to draw up a contrast, while rhetorically useful, leaves his claim open to challenges of authenticity. Therefore, by attending explicitly to his obligation to manage his claim with the use of a modal, and by being systematically vague about the particulars of what might have been, he is able to head off any attempts to undermine the claim.

Overall, Simon’s rhetorical work allows him to produce an account which answers the interviewer’s question concerning stroke-derived positives while positioning himself as the subject of an unfolding narrative, allowing him to carefully manage his accountability in making claims about potential outcomes absent his stroke. He attributes the change in the social arena as the means through which his stroke facilitated the rekindling of his friendship. He thus attends to identity aloneness by orienting to the post-stroke social arena as a protective factor. Alternatively, Extract 4 shows how this change has been problematic for Peter.

Extract 4:


1

PP:

regularly (.) and there’s a group of councillors being shown around the

2

the the err facilities

3

IV:

mhm

4

PP:

and one of them came up to me and says (.) ‘erm excuse me (.) I thought

5

there was a stroke group in there’ (.) I went ‘yes there is’ (.) he

6

says ‘why are you all laughing’ (1.0)

7

IV:

heh

8

PP:

that’s the sort of thing- that’s what you come across the prejudice you

9

come across (.) he [couldn’t understand that

10

IV:

    that’s the]

11

PP:

people with a stroke were having a good time

12

IV:

That’s a bizarre question to ask that (.) I think (.) [mhm

13

PP:

     but] (.)

14

when you’re on on this side on the survivor’s side you come you come-

15

you see it very regularly

16

IV:

mhm

17

PP:

they think we’re just in god’s waiting room (.) you [know just

18

IV:

     mhm ]

19

PP:

go away in the corner and don’t ig- and just get ignored .hh [till you]

20

pass away

21

IV:

 mhm ]

22

PP:

well not not anymore (1.0) that used to happen but (.) that’s not gonna

23

happen on my watch


Extract 4 consists of a narrative account which functions to example non-survivors’ treatment of Peter and his fellow survivors. It is situated in a community centre in which Peter had booked a room for his stroke support group meeting. Peter introduces the setting in lines 1–2 and follows up with an account of his discussion with a councillor wherein the legitimacy of the stroke group’s presence and authenticity of their category membership as survivors of stroke is challenged (ll. 4–6). A complex interplay between discursive devices rhetorically strengthens Peter’s claims about societal lack of understanding and prejudice. Foremost, the account in lines 4–6 itself makes use of reported speech, which adds credibility to the account by invoking the talk of another speaker, the councillor and functions to manage Peter’s own identity (and as such his ‘beliefs’) as being distinct from that of the councillor (Wiggins, 2017). Within the account, the councillor contrasts the emotive category of laughter with the category of stroke survivor and in doing so makes the inference that the stroke group’s entitlement to be there is established on false pretences. Instead of the councillor attributing this category incongruence to his own lack of understanding of stroke, he instead constructs it as something for which Peter has to account (‘why are you all laughing’), as if the stroke group were behaving improperly by laughing. Laughter is thus constructed as incompatible with the acceptable range of emotions that stroke survivors are entitled to experience, similar to how Simon in Extract 3 initially could not reconcile stroke with positivity. While this particular kind of category entitlement is unique to this study, the work of Whalen and Zimmerman (1990) on calls to emergency services illustrates the business of category entitlement in the realm of knowledge claims. Calls from the general public about criminal matters (e.g. the occurrence of a rape) were met with questions about the grounds of their claim from the operator, whereas calls from medical personnel about medical matters (e.g. an overdose) were treated as ‘fact’ and concluded swiftly. This adeptly illustrates the way in which the legitimacy to possess or act out certain kinds of psychological phenomena is strictly aligned to particular social categories.

Peter then goes on to treat this interaction as one iteration of a broader social problem, referring to it as ‘prejudice’ (l. 8) and a lack of understanding (ll. 9–11) from non-survivors. The interviewer responds in line 12 by assessing the councillor’s question as ‘bizarre’ and thus constructing it as unusual and uncommon, but Peter’s response from ‘the survivor’s side’ (l. 14) reconstructs it as something seen ‘very regularly’ (l. 15). In referring to ‘the survivor’s side’, Peter uses category entitlement to make prejudice against survivors, a problem exclusively understood by survivors. Second, the use of script formulation constructs the issue as something that ‘very regularly’ occurs and is thus a routine part of survivors’ everyday lives (Wiggins, 2017). Script formulation works rhetorically to assert that adversities on the survivors’ side are experienced constantly. Finally, consensus functions to rhetorically support Peter’s account of prejudice because, instead of relying on his individual experience, he recruits the experiences of all stroke survivors (e.g. ‘the survivor’s side’). Potter (1996) states that consensus supports accounts by constructing them as something real and ‘out there’, making therefore the claim of prejudice difficult to challenge.

Peter concludes his account by summarising non-survivors’ lack of understanding using the metaphor ‘they think we’re just in god’s waiting room’ (l. 17), in which there are several implicit meanings. It functions as an explanatory account for which the councillor’s confusion can be understood; how can one laugh with death imminent? Peter follows with another metaphor: ‘go away in the corner . . . and just get ignored .hh till you pass away’ (ll. 19–20). Together these metaphors construct a reality in which it is inconceivable that stroke survivors have any possibility of an enjoyable life and are instead resigned to being an inconvenience to society until death. Again, this is another example of a less-than-desirable subject position being offered up on behalf of society, one which Peter rejects by distinguishing himself as an agent on behalf of stroke survivors as a category; ‘well not not anymore (1.0) that used to happen but (.) that’s not gonna happen on my watch’ (ll. 22–23). In doing this he acts on behalf of all survivors in his capacity as a volunteer to resist the negative expectations of the social arena.

Overall, Peter orients to a social arena within which adversity is a natural, inevitable hurdle of post-stroke life. This social arena is constructed as offering up a position of passive acceptance of death as a sole choice, which Peter resists and instead takes up a position of agency. This contrasts against Simon’s positive account of the same social arena, demonstrating its inherent variety. In line with Haslam et al. (2008), Peter’s resistive stance is derived from his membership of the stroke survivor category, which acts as a protective factor against the harsh reality of post-stroke life.

## General conclusions

The analysis revealed that identity aloneness was attended to through the deployment of two broad rhetorical strategies. First, participants rejected the passive subject positions offered up to them by society by crafting and occupying their own positions of resilience. Second, participants managed the impact of the social world on their identities as stroke survivors. These two overarching rhetorical approaches governed the use of discursive devices which in turn provided the discursive resources necessary for survivors to attend to the issue of identity aloneness and account for how they were able to overcome it. The present study’s first contribution was thus to establish a discursive account for how the psychological business of identity aloneness is managed in interviews by stroke survivors. Hence, two promising concepts are opened up for further study: (1) that of the choice of resistance and (2) that of negotiation with one’s social world after stroke. Additionally, the present study serves to enrich the discursive literature by applying its principles to a novel issue (identity aloneness), related to the well-researched concept of identity, in a small group of stroke survivors (and their partners), a population who had not yet been examined by this approach. It further strengthens the case for the utility of discourse analysis by demonstrating that certain kinds of people have idiosyncratic ways of producing rhetorical accounts which are the means by which they discursively attend to issues of psychological significance (identity aloneness).

Importantly, these contributions should be examined in the context of the study’s limitations. The derivation of the findings from qualitative means which relied on a small sample are not generalisable. Our sample consisted of males, over 60 years old, all of whom were retired and had spouses/carers. These three participants covered a range of common impairments experienced by stroke survivors rather than representing an individual facet or specific sub-group of stroke. Additionally, none of our participants experienced speech and language difficulties because of their stroke. People with stroke and aphasia are particularly vulnerable to limited social support, negatively affecting physical recovery and increasing the likelihood of a second stroke. This should be taken at best as inspiration for further work across the varied nature of stroke (e.g. age, gender, specific comorbidity, time since injury, etc.) to further understand the specific differences that exist in experiences within these sub-groups.

Also, the use of interviews in discourse analysis is subject to tension among scholars, some of whom argue that talk emanating from the interview is an artefact of the setting, and hinders analysis of the psychological object of interest. Participants’ discursive choices may reflect their position as an interviewee, rather than what the analyst is interested in. However, gathering naturalistic data on this matter is ethically and practically problematic, given the private nature of issues like aloneness and loneliness, which may never be discussed spontaneously. Care should therefore be taken to identify everyday contexts suitable for a naturalistic analysis.

These limitations offer several avenues for further study. First, alternative qualitative methodologies, such as grounded theory (Glaser and Strauss, 2017), could be used to initiate the development of a complex account of the phenomenon of identity aloneness. This could make use of the present findings in developing a suitable interview schedule.

Conducting our interviews via video conferencing platforms removed restrictions often associated with face-to-face interviews such as travel time, expenses and participants distance from the research site. Although the use of online mediums for qualitative research is debated (see Thunberg and Arnell, 2022 for a review) for interviews with vulnerable groups such as stroke survivors, studies show that an online setting is predominantly positive (Weller, 2017), citing factors like physical distance between the researcher and participant facilitating greater connection and an increased sense of ease. Further work using discursive psychology could investigate the possibility of conducting data recording in a more naturalistic environment than in the present study including survivors’ homes or potentially less ethically problematic, in meetings with stroke support groups or health professionals..

Overall, the present study identified two rhetorical strategies that stroke survivors relied on to attend to the matter of identity aloneness. The findings advance both the stroke and the discursive literatures by further explicating the nascent concept of identity aloneness and applying a discursive methodology to a field in which it is seldom applied. It is suggested that further work continues to expand the theoretical account of identity aloneness in stroke and addresses the limitations of this study by investigating the possibility of collecting naturalistic data and by undertaking efforts to develop a quantitative paradigm of identity aloneness.

## Supplemental Material

sj-docx-1-hea-10.1177_13634593241279207 – Supplemental material for ‘They think we’re just in God’s waiting room’: A discursive study on identity aloneness in stroke survivors Supplemental material, sj-docx-1-hea-10.1177_13634593241279207 for ‘They think we’re just in God’s waiting room’: A discursive study on identity aloneness in stroke survivors by Lewis Jefferson and Stephen Dunne in Health

sj-docx-2-hea-10.1177_13634593241279207 – Supplemental material for ‘They think we’re just in God’s waiting room’: A discursive study on identity aloneness in stroke survivors Supplemental material, sj-docx-2-hea-10.1177_13634593241279207 for ‘They think we’re just in God’s waiting room’: A discursive study on identity aloneness in stroke survivors by Lewis Jefferson and Stephen Dunne in Health
