# Supplementary material for: ‘They think we’re just in God’s waiting room’: A discursive study on identity aloneness in stroke survivors
Source: Health (London). 2024 Sep 19;29(3):316–34. doi: 10.1177/13634593241279207 (PMC12049578; doi:10.1177/13634593241279207)
Supplement: sj-docx-1-hea-10.1177_13634593241279207 – Supplemental material for ‘They think we’re just in God’s waiting room’: A discursive study on identity aloneness in stroke survivors [file sj-docx-1-hea-10.1177_13634593241279207.docx]

As the first author, I conducted the interviews with participants, as well as the analysis and discussion. I conducted this study as part of a Master’s thesis and had no previous experience interacting with stroke survivors in a professional capacity, which the participants were aware of prior to interview. Although I felt the interviews were successful and that I built up a good rapport with the participants, it is likely that the profile of the interviews would have been slightly different with an interviewer more familiar with engaging with stroke survivors. On the one hand, participants may have felt more understood when interviewed by an individual whom they perceive to be an ‘expert’ in the phenomenology of stroke. On the other hand, my nascency to stroke may have functioned to attenuate any perceived power disparity between participant and interviewer. Given this, participants may have felt that they were under less pressure to give the ‘right answers’ during interviews, as there would be no reason that my claim to knowledge about the subject matter would be any more elevated than theirs.

In terms of the methodology, a number of decisions were influenced by my own interests. Having already produced well-received work (unpublished) utilising CDP, it is possible that my preference for CDP unconsciously informed its application in this study, where a thematic analytic approach might have been just as capable of elucidating the concept of identity aloneness given appropriate re-framing of the research question. Nevertheless, I still feel that my justification, based on the socially constructed and discursive properties of identity, necessitated the use of CDP. In contrast to reflexive thematic analysis, there is no homologue of Braun & Clarke’s (2006) 15 point checklist. However, Wiggins (2017) details the six ‘pitfalls’ of discursive psychology, which I believe afforded me the opportunity to conduct a sufficiently rigorous analysis with minimal personal bias.

Wiggins explicates six ways in which the integrity of a discursive analysis can be compromised. Pitfalls (1) and (6) are similar, consisting of under-analysis through summary of what is going on in the data, and identifying instances of discursive devices alone and passing that off as an analysis. Pitfall (2) consists of taking a moral stance on the data, introducing personal bias. Pitfall (3) is under-analysis through over-quotation; this is essentially letting the quotes speak for themselves without any attempt at interpretation. Pitfall (4) is the circular identification of discourses and patterns and appears when, in the pursuit of finding a discourse or pattern, an analyst uses a participant’s utterance to justify a claim about the data, and then presents the claim as the reason for the utterance, in a circular manner. This is seen in the analyst’s selection of cognitivist discourse (e.g., unhappy talk) as a means to evidence a participant’s mental state, and then use their mental state to explain why they engaged in unhappy talk, which contradicts the discursive psychological view of language. Finally, Pitfall (5) is the extrapolation of present findings to other settings with different contexts; an attempt to generalise findings, in other words.

Of these, given my experience with the methodology, I referred to these in my own analysis in order to preserve rigour, but often found that my instinct was already conducive to avoiding the pitfalls. The only occasion on which I had to consider them intently was under-analysis through taking sides, particularly when Peter recounted his interaction with the councillor during the stroke group meeting. My initial thoughts on the matter were apparent in the transcript where I refer to the councillor’s question as bizarre. However, I recognised that taking a moral stance during the analysis would have produced one that was sub-par, and so this pitfall reminded me to prevent doing so.
